# Symptomatic Intrathoracic Splenosis More than Forty Years After a Gunshot Injury

**DOI:** 10.7759/cureus.5985

**Published:** 2019-10-24

**Authors:** Adnan Khan, Sana Khan, Saran Pillai

**Affiliations:** 1 Critical Care, Freeman Health System, Joplin, USA; 2 Internal Medicine, Sindh Medical College, Karachi, PAK; 3 Emergency Medicine, Kerala Institute of Medical Sciences Hospital, Trivandrum, IND

**Keywords:** intrathoracic splenosis, splenosis, vats, ectopic spleen, trauma, chest pain, shortness of breath, splenectomy, malignancy mimic, thoracoscopic surgery

## Abstract

Thoracic splenosis is a rare heterotopic autotransplantation of the spleen into the thorax that occurs after trauma or surgery involving the spleen. It is most commonly found incidentally on imaging in the left hemithorax. To the best of our knowledge, only six symptomatic cases of thoracic splenosis have been described in the literature so far. We present a case of thoracic splenosis in a male with a remote history of a gunshot injury during childhood, who presented with chest pain and shortness of breath.

## Introduction

Ectopic spleen or splenosis is a condition that arises from the heterotopic autotransplantation of the spleen. The spleen will implant itself in another location, usually the serosal surface, deriving blood supply locally and progressing into differentiated nodules of splenic tissue [1]. This occurs most commonly after splenic rupture following trauma or surgeries. The incidence of splenosis ranges from 26% to 65% following trauma and up to 20% following elective splenectomy [2,3]. The most common sites of splenosis are within the abdominal and pelvic cavity, where it involves both the layers of the peritoneum. It is usually asymptomatic but can rarely mimic a peritoneal malignancy [4].

The thoracic cavity is a relatively rare location of splenosis most commonly found incidentally on imaging in the left pleural cavity [5]. It makes up about 18% of the total cases and is usually accompanied by a history of simultaneous rupture of the spleen and the diaphragm [6,7]. It commonly presents as variable-sized nodular lesions in the left thoracic cavity in an asymptomatic patient. Intrathoracic splenosis is usually managed expectantly unless the patient is symptomatic. However, in asymptomatic cases, a definitive diagnosis is most often not reached due to the rarity of this condition, and unnecessary surgical resection is done [8]​. We present a case of symptomatic intrathoracic splenosis occurring in a 51-year-old male who presented with chest pain and shortness of breath, 41 years after a gunshot injury wound. We stress the ability of symptomatic intrathoracic splenosis to mimic other medical conditions and to be a diagnostic dilemma for clinicians.

## Case presentation

A 51-year-old man presented to the emergency department with complaints of chest pain and shortness of breath for two weeks. Chest pain was intermittent, on the left side of the chest with no radiation, and aggravated by deep breathing. Shortness of breath was exertional in nature. The review of systems included weight loss of up to 15 pounds in the last four months. He denied any fevers, chills, night sweats, and sick contacts. He was a former smoker with a 40 pack-year history and a past medical history of hypertension and hyperlipidemia. Physical examination was within normal limits except for a scar on the chest, a remnant of a childhood gunshot injury that happened 41 years ago, when he was ten years of age.

Blood workup showed leukocytosis with white blood cell (WBC) count of 11700 cells per cubic millimeter, hemoglobin 14.3 gm/dl, and platelet count 306,000 per microliter. Renal function tests, serum electrolytes, and liver function tests were within normal limits. His electrocardiogram (EKG) showed some T-Wave inversions. Cardiac enzymes came back negative for any ischemia.

**Figure 1:**
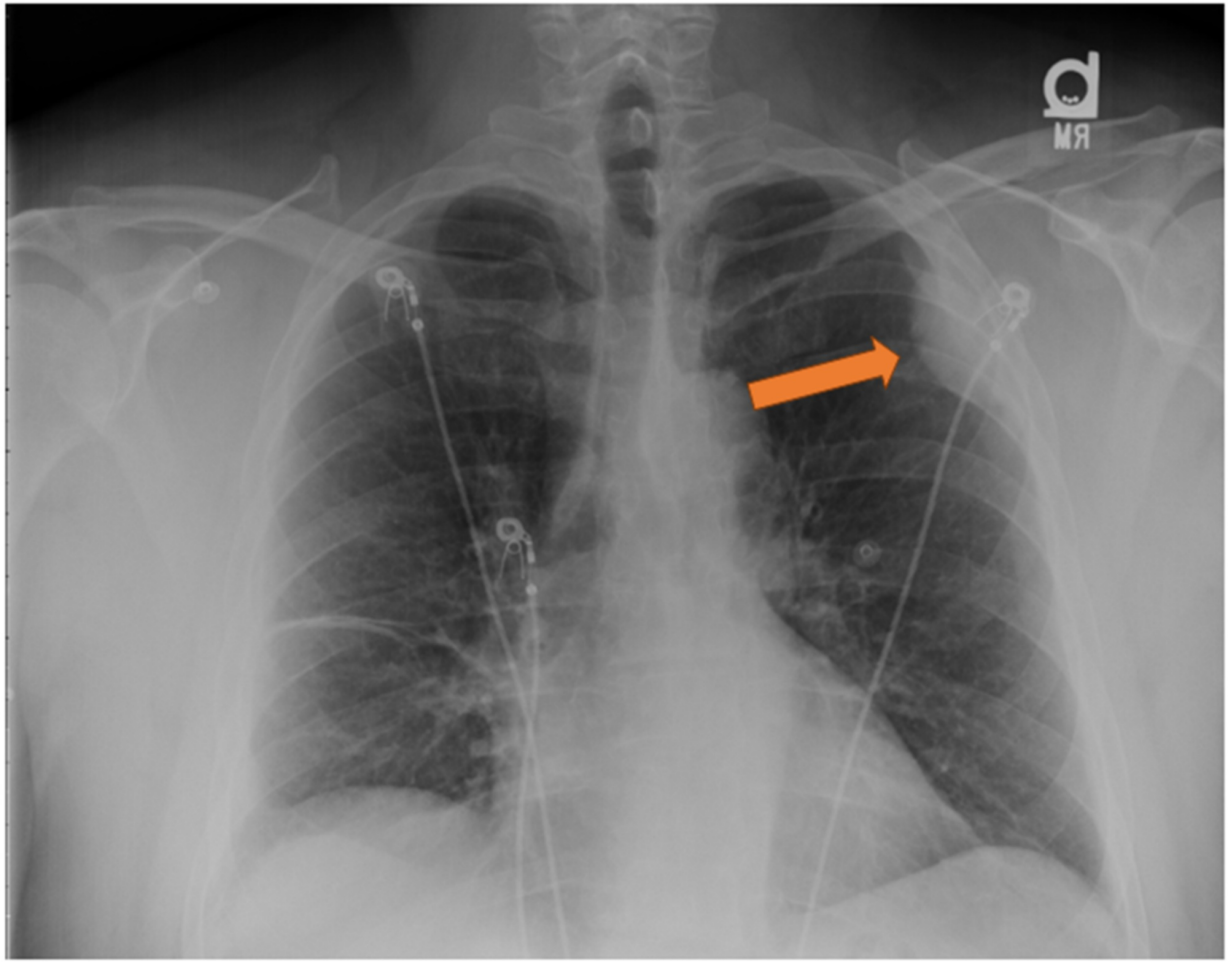
Chest X-ray, posteroanterior view Chest X-ray (posteroanterior view) showing peripheral opacity in the left upper lobe (orange arrow).

**Figure 2:**
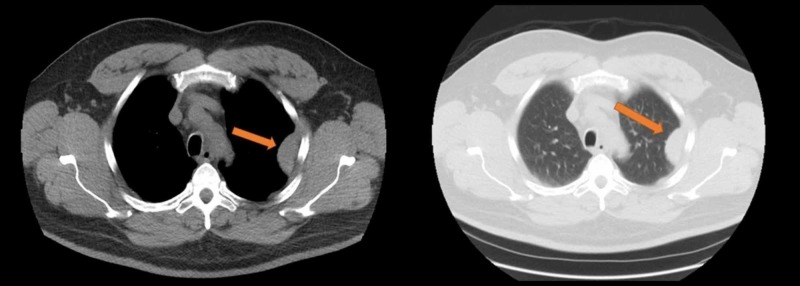
CT scan chest Computerized tomography (CT) scan of the chest showing a pleural-based left upper lobe mass (orange arrows).

**Figure 3:**
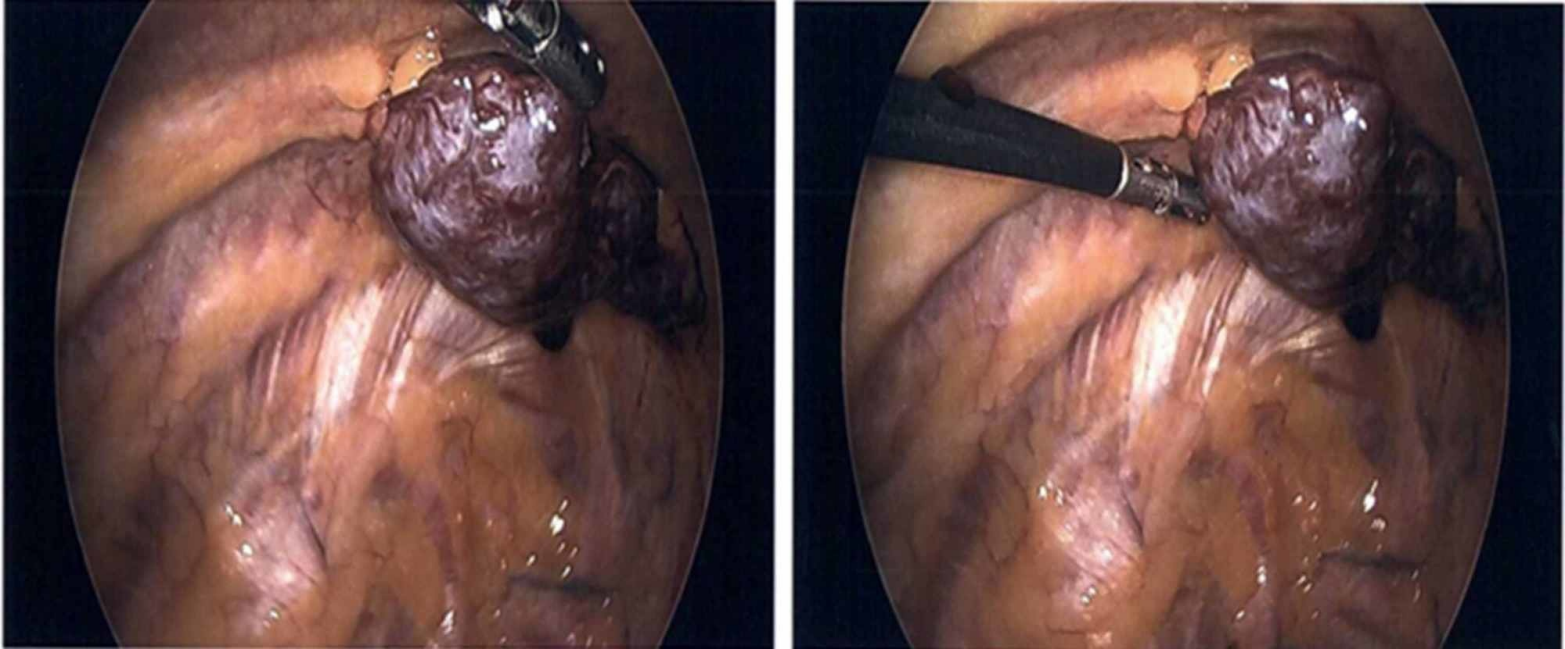
Video-assisted thoracoscopic (VATS) procedural capture VATS procedural capture showing lobulated mass along the chest wall.

His chest X-ray showed a peripheral opacity in the left upper lobe (Figure 1). Differentials included a pulmonary embolism/infarct or malignancy. Treatment was started for possible bronchitis, as he was admitted for further investigations. He underwent a computerized tomography (CT) scan of the chest which showed a pleural-based left upper lobe mass (Figure 2). However, CT-guided biopsy of the lung mass was non-diagnostic. Cardio-thoracic surgery was consulted, and he underwent video-assisted thoracoscopic (VATS) surgical resection of a lobulated mass along the left chest wall (Figure 3). Histopathology examination of the resected tissue at an outside facility came back positive for lymphoid tissue consistent with splenosis. Postoperative recovery was without incident, and the patient was discharged after a few days. The patient had no further complaints of chest pain or shortness of breath on repeat follow-up up to two months after the surgery.

## Discussion

The cases of splenosis reported in the literature have shown a steady rise with about 37 cases reported till 2003, and about 66 cases till 2010 [8,9]. Currently, about 81 cases have been reported in the literature so far. Splenosis is most commonly caused by either a penetrating or a blunt force trauma to the thoracoabdominal area. It has been observe in up to 66% of post-trauma splenectomy patients and in up to 65% of splenic rupture patients [5]. Within the abdomen, splenosis occurs in the peritoneum, omentum, and mesentery.

Most cases of intrathoracic splenosis occur concurrently with intraabdominal splenosis and rarely occur alone [5]. The majority of cases of intrathoracic splenosis are asymptomatic and are discovered incidentally on imaging as multiple nodules (75% cases) or solitary nodules (25% cases) [8,10]. When it occurs in the thorax, it most commonly occurs in the left pleural space and rarely within the left lung parenchyma [8]. To the best of our knowledge, there have been only six symptomatic patients with intrathoracic splenosis to date where the most common presenting complaints were cough, pleuritic chest pain, and hemoptysis [5,8,11,12]. Regional irritation or pressure has been cited as the cause of these symptoms.

The most common explanation for splenosis is the spillage of splenic pulp into adjacent body cavities during traumatic rupture; along with a diaphragmatic rupture in cases of intrathoracic splenosis. Other explanations include hematogenous seeding, hypoxic induction of tissue growth, and congenital ectopic cell rests, ​​​​​​all of which are supported by the incidence of splenosis without concomitant diaphragmatic rupture [5,13,14].

The most common means of diagnosing splenosis remains to be a percutaneous biopsy under CT guidance. However, a significant risk of bleeding exists, especially when biopsying splenic tissue. In addition, it can frequently be inconclusive as in our patient, or inaccessible or inaccurate, often being misdiagnosed as lymphoma [5,12,15]. Surgical resection through a thoracotomy or video-assisted thoracic surgery (VATS) has been increasingly followed because of the benefits of accurate histopathological diagnosis and the advantage of being therapeutic as well. Currently, several non-surgical options exist as well for the diagnosis of splenosis. Suspicion should be high when typical hematological markers of a splenectomy patient such as Howell-Jolly bodies or siderocytes are absent in the blood [8]. Ferumoxide-enhanced magnetic resonance imaging (MRI) provides a higher spatial resolution when visualizing reticuloendothelial tissue with a signal loss in the T2 sequence of the scan. Confirmation can also be made by radionuclide imaging using Tc-99m-labeled and heat damaged erythrocytes and measuring their splenic uptake, which is the gold standard method. A lesser sensitive method includes radionuclide imaging and measuring splenic uptake of Tc-99m sulfur colloid and indium-111-labeled platelets [8]. If the patient has a remote or recent history of trauma involving the spleen and suggestive imaging on CT or ultrasound, it is pertinent to proceed with the use of nuclear medicine studies instead of biopsy for confirmation.

Asymptomatic intrathoracic splenosis is managed conservatively according to current guidelines because of the procedural risk of surgery, and the slight risk of overwhelming infections resulting from decreased immunity [6,7,12]. There are mixed results regarding whether splenosis provides any measurable amount of immunity, but some studies have shown an improvement in phagocytosis in patients with splenosis [10,16]. Also, the splenic tissue is noninvasive and benign and shows an indolent growth pattern, and thus safe to be left alone. However, in practice, intrathoracic splenosis frequently gets resected because of the difficulty in reaching a preoperative diagnosis. Differentials include an intrathoracic malignancy (primary lung carcinoma, mesothelioma, non-Hodgkin's lymphoma, thymoma, neurogenic tumors or metastases), endometriosis [8].

Surgical intervention is indicated in all symptomatic patients and where the diagnosis is uncertain and where a possibility of malignancy exists. In our patient, the CT-guided biopsy results were unsatisfying which led to the decision to do surgical resection using VATS. Surgical management employing a thoracotomy approach is reserved for bigger or unapproachable nodules alone [8].

The mean duration between the occurrence of trauma and detection of thoracic splenosis is about 20 years. A gap of 44 years is the longest gap reported in the literature followed by 41 years in our patient [8]. This makes the diagnosis of intrathoracic splenosis very challenging. The index of suspicion should be high when either a relevant past history of trauma is present or when a physical exam shows scars of upper midline laparotomy or scars on the left upper quadrant [8]. Hence, a thorough history and physical examination become important to help decide whether to proceed with noninvasive nuclear radiographic studies and prevent unnecessary surgical resection.

## Conclusions

Thoracic splenosis is a rare anomaly that is commonly discovered incidentally on imaging. Physicians should have a high index of suspicion when there is a history of trauma or splenic surgery along with suggestive findings on imaging, in which case scintigraphy may be done for confirmation, instead of invasive biopsy or surgery. A thorough history and physical exam remains the key, especially since the detection of the nodule might occur decades after the injury has occurred. Surgical options should be considered in cases of symptomatic intrathoracic splenosis or when malignancy cannot be ruled out completely.
